# Serum Albumin Concentrations in Stable Chronic Obstructive Pulmonary Disease: A Systematic Review and Meta-Analysis

**DOI:** 10.3390/jcm10020269

**Published:** 2021-01-13

**Authors:** Elisabetta Zinellu, Alessandro G. Fois, Elisabetta Sotgiu, Sabrina Mellino, Arduino A. Mangoni, Ciriaco Carru, Angelo Zinellu, Pietro Pirina

**Affiliations:** 1 Unit of Respiratory Diseases, University Hospital Sassari (AOU), 07100 Sassari, Italy; elisabetta.zinellu@aousassari.it (E.Z.); agfois@uniss.it (A.G.F.); 2 Department of Medical, Surgical and Experimental Sciences, University of Sassari, 07100 Sassari, Italy; 3 Department of Biomedical Sciences, University of Sassari, 07100 Sassari, Italy; esotgiu@uniss.it (E.S.); sabrinamellino3@gmail.com (S.M.); carru@uniss.it (C.C.); azinellu@uniss.it (A.Z.); 4 Department of Clinical Pharmacology, College of Medicine and Public Health, Flinders University and Flinders Medical Centre, Adelaide 5001, Australia; arduino.mangoni@flinders.edu.au

**Keywords:** COPD, albumin, inflammation, oxidative stress, malnutrition

## Abstract

Background: Chronic obstructive pulmonary disease (COPD) is a progressive condition characterized by chronic airway inflammation and lung parenchyma damage. Systemic inflammation and oxidative stress also play a role in the pathogenesis of COPD. Serum albumin is a negative acute-phase protein with antioxidant effects and an important marker of malnutrition. The aim of this meta-analysis was to investigate differences in serum albumin concentrations between patients with stable COPD and non-COPD subjects. Methods: A systematic search was conducted, using the terms “albumin” and “chronic obstructive pulmonary disease” or “COPD”, in the electronic databases PubMed and Web of Science, from inception to May 2020. Results: Twenty-six studies were identified on a total of 2554 COPD patients and 2055 non-COPD controls. Pooled results showed that serum albumin concentrations were significantly lower in COPD patients (standard mean difference, SMD = −0.50, 95% CI −0.67 to −0.32; *p* < 0.001). No significant differences were observed in SMD of serum albumin concentrations between COPD patients with forced expiratory volume in the 1st second (FEV1) < 50% and those with FEV1 > 50%. Conclusions: Our systematic review and meta-analysis showed that serum albumin concentrations are significantly lower in patients with stable COPD compared to non-COPD controls. This supports the presence of a deficit in systemic anti-inflammatory and antioxidant defense mechanisms in COPD.

## 1. Introduction

Chronic obstructive pulmonary disease (COPD) is a common condition characterized by persistent respiratory symptoms and airflow limitation that is not fully reversible. The airflow limitation is caused by airway disease and/or lung parenchyma destruction [1]. COPD represents a major public health concern, with a global prevalence of 11.7% in 2010 [2], and it is now the third leading cause of death worldwide [3]. It is a complex disease resulting from the interaction between genetic predisposition and environmental factors. In particular, COPD is characterized by chronic inflammation due to exposure to tobacco smoke, the main risk factor for COPD, as well as to occupational exposures, outdoor and indoor air pollution, and childhood exposure to the above-mentioned risk factors [4]. Other risk factors, including lifestyle, diet, and physical activity, may also play a role [4]. This proposition is supported by the recent observation that many people develop the disease without smoking exposure [5]. Inhalation of cigarette smoke or other noxious particles causes lung inflammation that, in patients with COPD, is amplified and even further increased during acute exacerbations [6]. The mechanism responsible for the excess inflammatory response is not yet understood, although oxidative stress may play an important role [7]. Oxidants contained in cigarette smoke and in other inhaled particulates, as well as those produced by activated inflammatory cells, might overcome the antioxidant defense systems, leading to oxidative stress. Human serum albumin (HSA) is a multifunctional plasma protein that accounts for over 50% of the total plasma protein content. Physiologically, HSA exists predominantly in a reduced state containing a free cysteine residue (Cys34) [8]. This residue constitutes the largest pool of thiols in the peripheral blood and represents the main pathway through which albumin scavenges reactive oxygen and nitrogen species (ROS and RNS) [9,10]. In addition to its antioxidant properties, albumin is also a negative acute phase reactant, and its concentrations decrease during the acute phase response [11]. Moreover, albumin is an established clinical marker of malnutrition [12]. Malnutrition is common among COPD patients and negatively impacts on quality of life, risk of exacerbations, length of hospital stay, and overall healthcare costs [13]. Similarly, hypoalbuminemia has been associated with a prolonged length of hospital stay during acute exacerbations, acute respiratory failure, and increased mortality in patients with COPD [14,15,16]. Several studies have investigated serum albumin concentrations in patients with stable COPD and non-COPD subjects, with some conflicting results. We sought to address this issue by conducting a systematic review and meta-analysis in order to investigate the presence and the effect size of differences in serum albumin concentrations between the two groups.

## 2. Experimental Section

### 2.1. Search Strategy, Eligibility Criteria, and Study Selection

A systematic search, using the terms “albumin” and “chronic obstructive pulmonary disease” or “COPD”, was conducted in the electronic databases PubMed and Web of Science from inception to May 2020. The references of the retrieved articles and reviews were also cross-checked to identify additional studies. Eligibility criteria were as follows: (i) assessment of serum or plasma albumin; (ii) comparison of subjects with or without COPD (case-control design); (iii) >10 subjects investigated; and (iv) articles in English. Two investigators (ES and SM) independently screened individual abstracts. If relevant, they independently reviewed the full articles; any disagreement was resolved by a third investigator (AZ). The Newcastle–Ottawa scale was used to assess the quality of each study by evaluating the following components: cohort selection, cohort comparability on the basis of the design or analysis, how the exposure was determined, and how the outcomes of interest were evaluated [17]. Studies achieving a score of six or more were considered to be of high quality.

### 2.2. Statistical Analysis

Standardized mean differences (SMD) were used to build forest plots of continuous data and to evaluate differences in serum albumin concentrations between non-COPD controls and COPD patients. A *p*-value < 0.05 was considered statistically significant, and 95% confidence intervals (CIs) were reported. In two studies [18,19], the median and interquartile range (IQR) values were extracted from a graph by the Graph Data Extractor program, and the mean and standard deviation values were calculated from the median and IQR, as previously described [20]. Q statistic was used to test the heterogeneity of SMD across studies (significance level at *p* < 0.10). Inconsistency across studies was evaluated through the I^2^ statistic (I^2^ < 25%, no heterogeneity; I^2^ between 25% and 50%, moderate heterogeneity; I^2^ between 50% and 75%, large heterogeneity; and I^2^ > 75%, extreme heterogeneity) [21,22]. A random-effects model was used to calculate the pooled SMD and corresponding 95% confidence intervals, due to high heterogeneity. The influence of each individual study on the overall risk estimate was investigated through sensitivity analysis, by sequentially excluding one study in each turn [23]. The associations between study size and magnitude of effect were analyzed by means of Begg’s adjusted rank correlation test and Egger’s regression asymmetry test at the *p* < 0.05 level of significance to assess the presence of potential publication bias [24,25]. We also performed the Duval and Tweedie “trim and fill” procedure [26] to further test the possible effect of publication bias. This method recalculates a pooled SMD by incorporating the hypothetical missing studies as though they actually existed, to augment the observed data so that the funnel plot is more symmetric. Statistical analyses were performed using Stata 14 (STATA Corp., College Station, TX, USA). The study was fully compliant with the PRISMA statement for reporting of systematic reviews and meta-analyses [27].

## 3. Results

**Figure 1:**
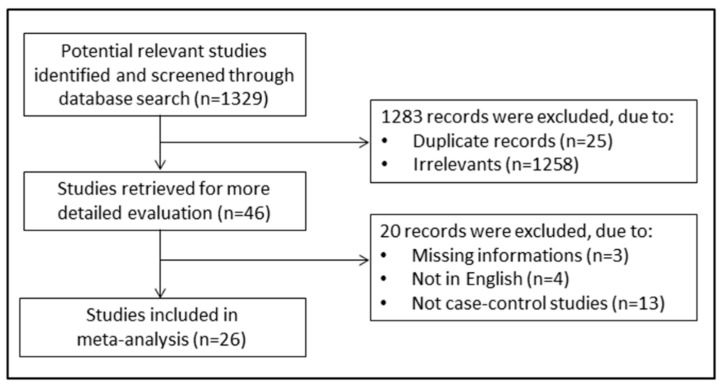
Flow chart of study selection.

**Figure 2:**
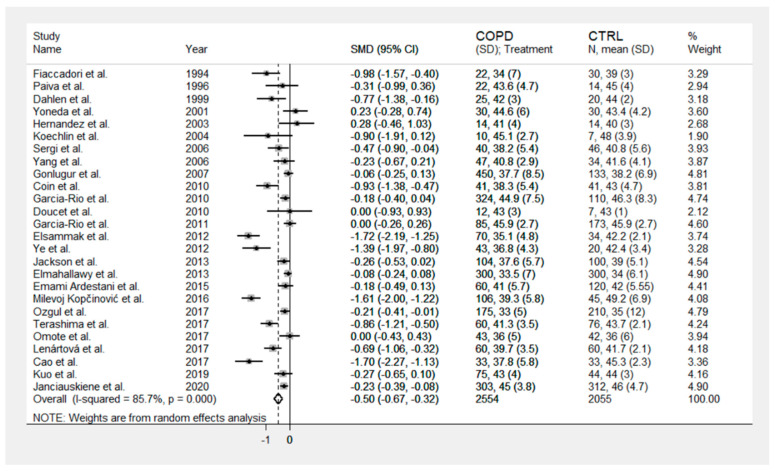
Forest plot of studies examining serum albumin in chronic obstructive pulmonary disease (COPD).

**Figure 3:**
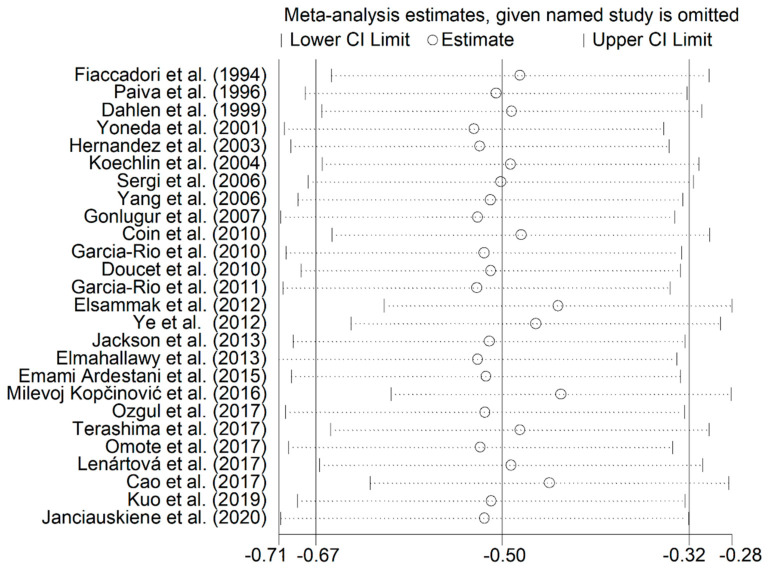
Sensitivity analysis of the association between albumin and COPD. The influence of individual studies on the overall standardized mean difference (SMD) is shown. The middle vertical axis indicates the overall SMD, and the two vertical axes indicate the 95% confidence intervals (CI). Hollow circles represent the pooled SMD when the remaining study is omitted from the meta-analysis. Two ends of each broken line represent 95% CI.

**Figure 4:**
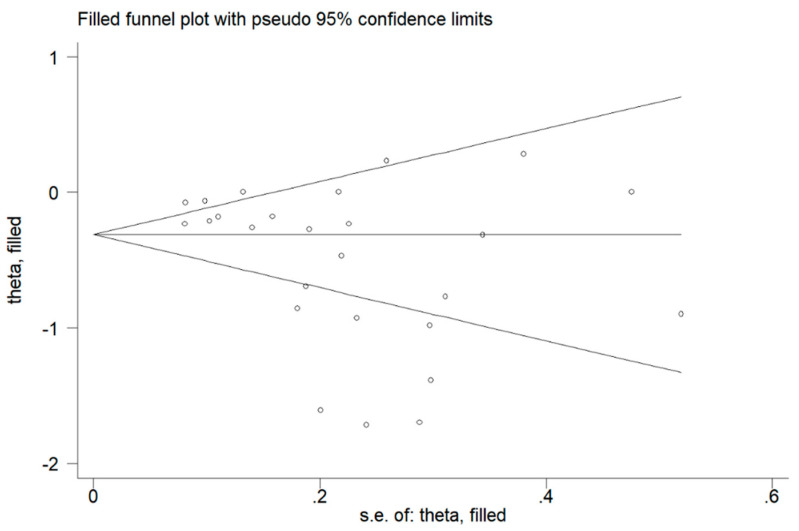
Funnel plot of studies investigating non-COPD controls and patients with COPD after trimming and filling. Dummy studies and genuine studies are represented by enclosed circles and free circles, respectively.

A flow chart depicting the screening process is described in Figure 1. We initially identified 1329 studies. A total of 1283 studies were excluded because they were either duplicates or irrelevant. After a full-text revision of 46 articles, 20 studies were excluded because they did not meet the inclusion criteria. Thus, twenty-six studies were included in the meta-analysis [18,19,28,29,30,31,32,33,34,35,36,37,38,39,40,41,42,43,44,45,46,47,48,49,50,51]. The characteristics of the retrieved studies, published between 1994 and 2020, are presented in Table 1. A total of 2554 COPD patients (74% males) and 2055 non-COPD controls (63% males) were evaluated. Overall, the mean age of participants across all studies was 61.7 years in COPD patients and 64.6 years in controls. Almost all (24 out of 26) were prospective studies. In sixteen studies, the diagnosis of COPD was made according to the Global Obstructive Lung Disease (GOLD) guidelines, whilst the American Thoracic Society (ATS) guidelines were used in five, the European Respiratory Society (ERS) guidelines were used in four, and the Spanish COPD Guidelines (GesEPOC) were used in one. Significant differences in serum albumin concentrations between COPD patients and control subjects were reported in 50% of the selected studies. The forest plot for serum albumin concentrations in COPD patients and controls is shown in Figure 2. Due to the extreme heterogeneity between studies (I^2^ = 85.7%, *p* < 0.001), random-effects models were used to perform the analysis. Pooled results showed that serum albumin concentrations were significantly lower in COPD patients (SMD = −0.50, 95% CI −0.67 to −0.32; *p* < 0.001). Sensitivity analysis, reported in Figure 3, showed that the effect size was not modified when any single study was in turn removed (effect size ranged between −0.44 and −0.52). The Begg’s (*p* = 0.04) and Egger’s tests (*p* = 0.01) showed a significant publication bias; however, the trim-and-fill analysis found that no study was missing or should be added (Figure 4).

In 23 studies, the control group comprised healthy subjects. In the remaining three, it consisted of subjects hospitalized for other medical conditions or some type of surgery [28], patients with diseases other than COPD [43], or lung cancer patients [48]. The SMD was not affected (−0.53, 95% CI −0.72 to −0.33, *p* < 0.001; I2 = 86.1%, *p* < 0.001) after removing these studies from the meta-analysis.

**Figure 5:**
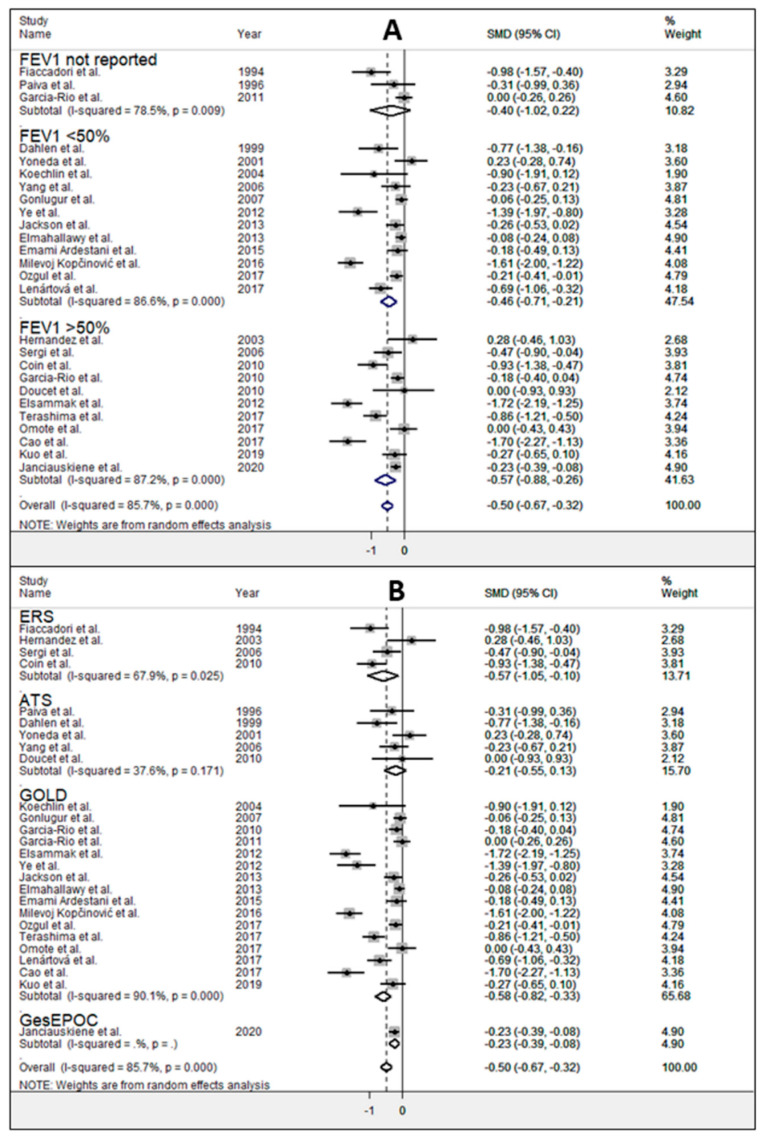
Forest plot of studies examining albumin and COPD according to disease severity evaluated by FEV_1_ (**A**) and guidelines used for the diagnosis (**B**).

We also investigated the effects of disease severity (forced expiratory volume in the 1st second FEV1 < 50% or FEV1 > 50%) and the guidelines used for diagnosis. No significant differences (*p* = 0.73) were observed in SMD of serum albumin concentrations between subjects with FEV1 < 50% (SMD = −0.46, 95% CI −0.71 to −0.21, *p* < 0.001; I^2^ = 86.6%, *p* < 0.001) and those with FEV1 > 50% (SMD = −0.57, 95% CI −0.88 to −0.26 g/L, *p* < 0.001; I^2^ = 87.2%, *p* < 0.001) (Figure 5A). Analysis based on specific guidelines (Figure 5B) also indicated similar pooled SMD values between studies recruiting by ERS (SMD = −0.57, 95% CI −1.05 to −0.10, *p* = 0.017) and GOLD guidelines (SMD = −0.58, 95% CI −0.82 to −0.33, *p* < 0.001) with a slight improvement in heterogeneity that may be classified as large in the ERS group (I^2^ = 67.9%, *p* = 0.025) and extreme in the GOLD group (I^2^ = 90%, *p* < 0.001). In the sub-group of studies using ATS guidelines for COPD diagnosis, we found that serum albumin concentrations were not significantly different between COPD patients and control subjects (SMD = −0.21, 95% CI –0.55 to 0.13, *p* < 0.124; I2 = 37.6%, *p* < 0.171). Then, we investigated age, sex, body mass index (BMI), FEV1, FEV1/forced vital capacity (FVC), C-reactive protein (CRP), and continent where the study was conducted (Europe, Africa, Asia, and America) and publication year as possible contributors to between-study variance. However, none of these variables was found to be significantly related to pooled SMD by meta-regression analysis (age (*t* = −0.81, *p* = 0.429), sex (*t* = 0.08, *p* = 0.940), BMI (*t* = 1.37, *p* = 0.187), FEV1 (*t* = 0.04, *p* = 0.965), FEV1/FVC (*t* = 0.55, *p* = 0.591), CRP (*t* = −0.60, *p* = 0.560), continent (*t* = 0.87, *p* = 0.393) and publication year (*t* = −1.18, *p* = 0.250)).

## 4. Discussion

COPD is characterized by the presence of persistent respiratory symptoms and a progressive airflow limitation caused by exposure to noxious particles. The GOLD definition of COPD focuses on the association of the airflow limitation with the inflammatory response of the lung that, in patients with COPD, seems to be altered [1]. Inflammation in COPD is characterized by excess alveolar macrophages, neutrophils, and T lymphocytes that secrete a variety of proinflammatory mediators, including cytokines, chemokines, and growth factors [6]. Oxidative stress is thought to play a key role in driving COPD-related inflammation. In fact, both exogenous ROS and those released by activated inflammatory and structural cells might lead to the activation of proinflammatory molecules, to lipid, protein, and DNA damage, and to corticosteroid resistance through the inactivation of histone deacetylase 2 [6]. This prevents corticosteroids from inactivating inflammatory genes. Moreover, oxidative stress suppresses nuclear factor erythroid 2-related factor 2 (Nrf2) activity, resulting in reduced antioxidant gene expression [52]. In addition, COPD patients exhibit a systemic proinflammatory state characterized by an increase in circulating cytokines, chemokines, and acute-phase proteins [53]. Moreover, in these patients, the pro-oxidant mechanisms overcome the antioxidant defenses with consequent oxidative stress [12,54]. Albumin is a multifunctional plasma protein that is described to diminish in inflammatory conditions, and it is considered a negative acute-phase protein [11]. Moreover, albumin has important antioxidant properties being the major extracellular source of sulfhydryl groups, which represents an important first defense system against oxidative stress [55]. We have recently reported a reduction in plasma protein sulfhydryl groups in COPD patients compared to healthy controls [56]. In addition, reduced levels of antioxidants have been extensively reported in patients with COPD, as mentioned before [54]. Another important role of albumin is that of a clinical biomarker of malnutrition [12]. Hypoalbuminemia can be caused by poor food intake/absorption, older age, the presence of comorbidities, and proinflammatory cytokines that inhibit albumin production. The influence of these factors is accumulative on the risk of hypoalbuminemia [57]. In patients with COPD, these factors often co-exist. For example, the prevalence of COPD increases with age, peaking after sixty years of age [1]. Moreover, it frequently occurs in association with several comorbidities [58,59] and with the progressive decline in exercise capacity, due to skeletal muscle wasting in older people. In turn, this is due to an imbalance between protein synthesis and breakdown. Malnutrition and weight loss are common among people with COPD and certain metabolic characteristics, such as a depleted fat mass and fat-free mass, can further impact on the loss of lung function capacity [13,60]. These features may be observed in stable COPD irrespectively of the degree of airflow limitation [61]. Our meta-analysis showed that overall, serum albumin concentrations were significantly lower in COPD patients compared to non-COPD controls. However, there was significant heterogeneity and publication bias, even if trim-and-fill analysis found that no study was missing or should be added. Age, sex, BMI, FEV1, FEV1/FVC, CRP, the continent where the study was conducted, and the year of publication did not contribute to this heterogeneity or to the pooled SMD. Conversely, the different guidelines used for COPD diagnosis contributed to the observed heterogeneity. We were not able to consider other factors, as they were not discussed in individual studies. These factors include the proportion of former and current smokers, the use of specific medications, and pre-analytical and analytical factors, such as sample storage, timing, conditions, and procedures for analysis. Moreover, three studies included in the meta-analysis comprised non-healthy subjects in the non-COPD group. Although albumin concentrations might have been theoretically affected, the SMD remained virtually unchanged after removing these three studies. Another limitation is represented by the relatively small number of subjects in a significant number of studies, which limits the representativity of the sample in terms of variability in disease phenotype and severity. This might have also explained the lack of significant differences after sorting for disease severity. Despite these limitations, our meta-analysis has the merit of comprehensively reviewing, for the first time, the available evidence on this topic, serving as a robust reference for future studies. In conclusion, our review and meta-analysis showed that serum albumin concentrations were significantly lower in patients with stable COPD compared to non-COPD subjects. This supports the presence of a deficit in systemic anti-inflammatory and antioxidant defense mechanisms, as well as malnutrition, in COPD. However, the significant heterogeneity of the studies assessed warrants further investigations with rigorous methods and standardized diagnostic criteria.

## Figures and Tables

**Table 1:** Summary of the studies on COPD vs. controls included in the meta-analysis.

				Control Group				COPD Group			
First Author, Year Country	Study Design	Diagnosis	NOS (Stars)	*n*	Age (Years)	Gender (M/F)	Albumin Mean ±SD	*n*	Age (Years)	Gender (M/F)	Albumin Mean ± SD
Fiaccadori, 1994 Italy, [28]	P	ERS	7	30	60	25/5	39 ± 3	22	63	19/3	34 ± 7
Paiva, 1996 Brazil, [29]	P	ATS	7	14	52.8	14/0	45 ± 4	22	59.4	22/0	43.6 ± 4.7
Dahlen, 1999 Sweden, [30]	P	ATS	6	20	66	4/16	44 ± 2	25	42	15/10	42 ± 3
Yoneda, 2001 Japan, [31]	P	ATS	7	30	64	29/1	44.6 ± 6.0	30	64	29/1	43.4 ± 4.2
Hernandez, 2003 Spain, [32]	P	ERS	6	14	59	14/0	40 ± 3	14	64	14/0	41 ± 4
Koechlin, 2004 France, [33]	P	GOLD	7	7	60	7/0	48 ± 3.9	10	58	10/0	45.1 ± 2.7
Sergi, 2006 Italy, [35]	P	ERS	7	46	77.7	46/0	40.8 ± 5.6	40	75.7	40/0	38.2 ± 5.4
Yang, 2006 China, [34]	P	ATS	7	34	64.8	34/0	41.6 ± 4.1	47	67.6	47/0	40.8 ± 2.9
Gonlugur, 2007 Turkey, [36]	R	GOLD	6	133	60.9	103/30	38.2 ± 6.9	450	61.9	348/102	37.7 ± 8.5
Coin, 2010 Italy, [37]	P	ERS	7	41	76	41/0	43 ± 4.7	41	75.7	41/0	38.3 ± 5.4
Garcia-Rio, 2010 Spain, [18]	P	GOLD	7	110	55	51/59	46.3 ± 8.3 ^§,^*	324	64	241/83	44.9 ± 7.5 ^§,^*
Doucet, 2010 Canada, [39]	P	ATS	6	7	60	2/5	43 ± 1	12	60	8/4	43 ± 3
Garcia-Rio, 2011 Spain, [38]	P	GOLD	7	85	--	--	45.9 ± 2.7	173	--	--	45.9 ± 2.7
Elsammak, 2012 Saudi Arabian, [40]	P	GOLD	7	34	63.0	20/14	42.2 ± 2.1	70	62.6	47/23	35.1 ± 4.8
Ye, 2012 China, [41]	P	GOLD	8	20	67.5	15/5	42.4 ± 3.4	43	68.4	40/3	36.8 ± 4.3
Jackson, 2013 England, [42]	P	GOLD	9	100	63	59/41	39.0 ± 5.1	104	65	60/44	37.6 ± 5.7
Elmahallawy, 2013 Egypt, [43]	P	GOLD	7	300	64.7	138/162	34.0 ± 6.1	300	65	152/148	33.5 ± 7
Emami Ardestani, 2015 Iran, [44]	P	GOLD	6	120	43.7	120/0	42 ± 5.55	60	59.1	60/0	41 ± 5.7
Milevoj Kopčinović, 2016 Croatia, [45]	P	GOLD	7	45	58.0	20/25	49.2 ± 6.9	106	64.3	80/26	39.3 ± 5.8
Ozgul, 2017 Turkey, [46]	P	GOLD	9	210	57.4	119/91	35 ± 12	175	61	110/65	33 ± 5
Terashima, 2017 Japan, [47]	P	GOLD	6	76	65.1	49/27	43.7 ± 2.1	60	73.9	55/5	41.3 ± 3.5
Lenártová, 2017 Slovakia, [19]	P	GOLD	7	60	--	--	41.7 ± 2.1 ^§,^*	60	--	--	39.7 ± 3.5 ^§,^*
Omote, 2017 Japan, [48]	R	GOLD	7	42	63	32/10	36 ± 6	43	67	37/6	36 ± 5
Cao, 2017 China, [49]	P	GOLD	6	33	70	26/7	45.3 ± 2.3	33	73	26/7	37.8 ± 5.8
Kuo, 2019 Taiwan, [50]	P	GOLD	6	44	53.3	36/8	44 ± 3	75	71.5	67/8	43 ± 4
Janciauskiene, 2020 Spain, [51]	P	GesEPOC	7	312	55	138/174	46 ± 4.7	303	64	223/80	45 ± 3.8

* Mean and standard deviation (SD) were estimated from formulas using the median and range as described by Wan et al. [20]. ^§^ Values extrapolated by Graph Data Extractor; NOS: Newcastle–Ottawa quality assessment scale for case-control studies. P = prospective; R = retrospective.

## Data Availability

No new data were created or analyzed in this study. Data sharing is not applicable to this article.
